# Isolation and Characterization of a Heparin-Like Compound with Potent Anticoagulant and Fibrinolytic Activity from the Clam *Coelomactra antiquata*

**DOI:** 10.3390/md18010006

**Published:** 2019-12-19

**Authors:** ZhenXing Du, XueJing Jia, Jing Chen, SiYi Zhou, JianPing Chen, XiaoFei Liu, XiaoHuang Cao, SaiYi Zhong, PengZhi Hong

**Affiliations:** 1 School of Food Science and Technology, Guangdong Ocean University, Zhanjiang 524088, China; 18320375284@163.com (Z.D.); jiaxj@gdou.edu.cn (X.J.); 15292604455@163.com (J.C.); ssuyi99@163.com (S.Z.); cjp516555989@126.com (J.C.); liuxf169@126.com (X.L.); caoxhfood@163.com (X.C.); hongpengzhi@126.com (P.H.); 2 Shenzhen institute, Guangdong Ocean University, Shenzhen 518108, China; 3 Guangdong Provincial Key Laboratory of Aquatic Products Processing and Safety, Guangdong Province Engineering Laboratory for Marine Biological Products, Zhanjiang 524088, China; 4 Collaborative Innovation Center of Seafood Deep Processing, Dalian Polytechnic University, Dalian 116034, China

**Keywords:** shellfish, heparin, structural characteristics, anticoagulant activity, fibrinolytic activity

## Abstract

Heparin from mollusks with unique sulfated glycosaminoglycan exhibits strong anti-thrombotic activities. This study reports on a purified heparinoid from *Coelomactra antiquata*, which shows potent anticoagulant and fibrinolytic abilities. Its structure was characterized by infrared spectroscopy, high-performance liquid chromatography, and one-dimensional and two-dimensional nuclear magnetic resonance spectroscopy. Its fibrinolytic activity was determined in vitro and in vivo. Its anticoagulant activity was determined by activated partial thromboplastin time (APTT), prothrombin time (PT), and thrombin time (TT). The results indicated that clam heparinoid was a homogeneous glycosaminoglycan with a molecular weight of 30.99 kDa, mainly composed of →4)-α-IdoA2S-(1→4)-α-GlcNS3S6S (or GlcNS6S)-(1→4)-β-GlcA-(1→4)-α-GlcNS6S (or GlcNAC)-(1→. Furthermore, this heparinoid showed a highly anticoagulant titer and fibrinolytic value of 149.63 IU/mg and 1.96 IU/mg, respectively. In summary, clam heparinoid shows great potential for application in the clinic and antithrombotic drugs industry.

## 1. Introduction

Heart and vascular diseases, including thrombosis, are the leading causes of death in the United States and Europe. Even though, after the introduction of antithrombotic agents, particularly heparin and its derivatives, the incidence of deadly vascular diseases has decreased substantially (about 30%). But when compared with malignant cancers, they are still the main cause of death. This explains the efforts to discover and develop specific and more potent antithrombotic agents [1].

**Figure 1:**
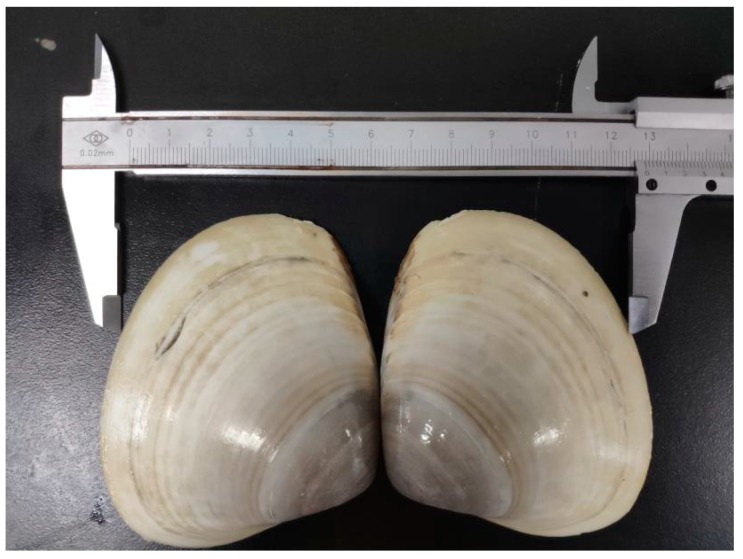
*Coelomactra antiquata* from Zhanjiang Dongfeng Market, China.

As a costly and nutrient-rich seafood shellfish, *Coelomactra antiquata* has attracted a lot of attention in China (Figure 1). Additionally, according to the Compendium of Materia Medica, *C. antiquata* has been used as a tonifying essence for lung moistening. Wen et al. reported that the use of *C. antiquata* water extract at doses of 50–150 mg/kg could significantly reduce the blood sugar concentration in diabetic mice, and Yang et al. reported that the inhibitory rate of *C. antiquata* polysaccharides at a dose of 30 mg/kg on human esophageal squamous cell carcinoma EC-9706 cells in nude mice was 28.85% [2,3]. However, there have been no reports on heparins from *C. antiquata*.

Heparin, originally found in dog liver, is a glycosaminoglycan that is mainly composed of L-iduronic acid (IdoA), D-glucuronic acid (GlcA), α-D-glucosamine (GlcN), and its derivatives (acetylation, sulfation) [4]. Heparin has been used in clinics for more than 50 years because of its potent anticoagulant ability. It also possesses strong anti-asthma, anti-proliferation, and anti-thrombosis activities [5,6,7]. Heparin is obtained from pig intestine and bovine lung for commercial use. However, this process is considered high risk among groups with different religious beliefs and regarding lethal diseases and quality control [8]. So, there is high demand for finding novel and safe heparin sources. 

Mollusk heparin has received more attention because of its available raw materials, convenient isolation, and potent biological activities. It could be an alternative, since heparin-like compounds are widely present in bivalves. Some mollusks contain compounds very similar to mammalian heparin with a high affinity for antithrombin, while others are composed of very unusual disaccharide units [9]. Gomes et al. obtained a heparin-like glycan from the mollusk *Nodipecten nodosus* that was shown to inhibit the activation of endothelial cells and the aggregation of inflammatory cells in mice with fatty peritonitis. Arumugam et al. reported the anti-proliferative effect of 1–100 μg/mL giant clam (*Tridacna maxima*) heparin and green mussel (*Perna viridis*) heparin on pulmonary artery smooth muscle cells. Dreyfuss et al. extracted heparin from a marine shrimp and showed significant anti-angiogenesis activity at 4.5~450 ng/mL [10,11,12]. Brito et al. reported the anti-inflammatory properties of a heparin-like compound from the shrimp *Litopenaeus vannamei*. This compound reduced the activity of matrix metalloproteinase (MMP-9) secreted by human activated leukocytes by almost 90%. Besides, the negligible bleeding potential makes this compound a better alternative than mammalian heparin as a possible anti-inflammatory drug [13]. Heparin and hybrids with heparan sulfate are also found in ascidians [14], crabs [15], and sea urchins [16]. In spite of their low anticoagulant activity, some display remarkable antithrombotic activity. Heparin isolated from an ascidian showed antithrombotic activity in an arterial animal model comparable to mammalian heparin, but with lower bleeding effects [17].

One of the most important aspects of heparin extracted from marine sources is the low-level risk of contamination with microorganisms like viruses and bacteria, as marine organisms are evolutionally secluded from terrestrial mammals [18]. Besides, a large number of studies show that the side effects of heparin/heparin analogues are not obvious when compared with mammalian heparin [10,11,13,15,19,20], which may reduce the bleeding risk of patients. 

Although many studies have been carried out on marine heparin [21,22,23], the structural features and bioactivities of *C. antiquata* heparin remain unclear. The aim of this study was to isolate heparin from *C. antiquata*, characterize its structure, and evaluate its antithrombotic effects by analyzing its anticoagulant and fibrinolytic activities. This study provides a theoretical foundation for the further development and utilization of *C. antiquata* heparin.

## 2. Results

### 2.1. Heparinoid Purified from C. antiquata

**Figure 2:**
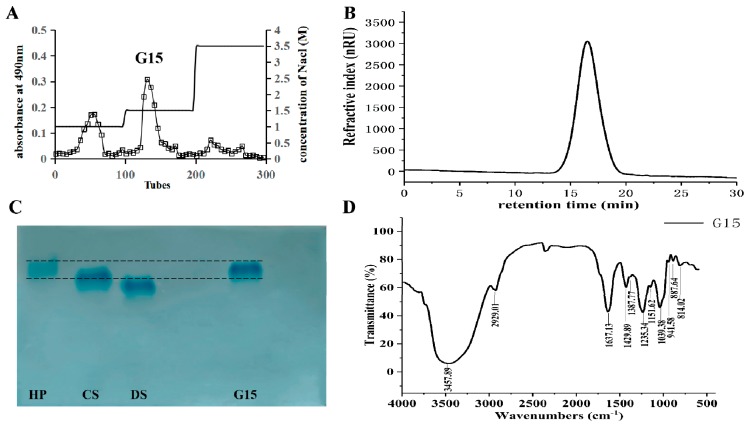
Purification, HPGPC, electrophoresis, and IR spectrum of the clam heparinoid (G15). (**A**) *C. antiquata* heparinoid was purified with AMBERLITE FPA98 CI and eluted with 1, 1.5, and 3.5 M NaCl. The fractions eluted with 1.5 M NaCl were collected and designated as G15; (**B**) HPGPC chromatogram of G15; and (**C**) cellulose acetate electrophoresis of G15. Reference substances: Mammalian heparin (HP), Chondroitin sulfate (CS), Dermatin sulfate (DS). (**D**) Infrared spectrum of G15.

A natural heparinoid was obtained from the clam *C. antiquata* by enzymatic hydrolysis, ion exchange, and ethanol precipitation. G15 was eluted with 1.5 M NaCl, and the yield of G15 was 1.17 mg/g (dry weight) (Figure 2A). A symmetrical single peak appeared in the high-performance gel permeation chromatography (HPGPC) chromatogram result (Figure 2B), indicating that G15 is a homogeneous compound with a molecular weight of 30.99 kDa. It has been reported the crab heparinoid molecular weight is 19 kDa, that of porcine heparin is 16 kDa, and that of bovine heparin is 25 kDa [15]. In contrast, the G15 molecular weight is higher than the heparin values mentioned above, which might be due to their different raw materials [1]. In addition, G15 showed a single band and similar electrophoretic mobility (Figure 2C), indicating that G15 is a purified fraction.

The infrared spectrum of G15 is shown in Figure 2D. The big and broad peak at 3457.89 cm^−1^ corresponds to the O–H stretching vibration, and the signal at 2929.01 cm^-1^ corresponds to the C–H stretching vibration [24]. The signal at 1637.13 cm^-1^ is caused by the N–H angular vibration of an amino group, the signal at 1429.89 cm^−1^ is attributed to the C–O stretching vibration of the carboxyl group, and the signal at 1235.34 cm^−1^ is due to the S=O stretching vibration of the sulfate group. The band at 1039.38 cm^-1^ is corresponds to the stretching vibration of C–O–C and -OH of the pyran ring, and the signal at 887.64 cm^-1^ indicates that G15 contains a β-glycosidic bond [25].

### 2.2. Monosaccharide Composition Analysis

**Figure 3:**
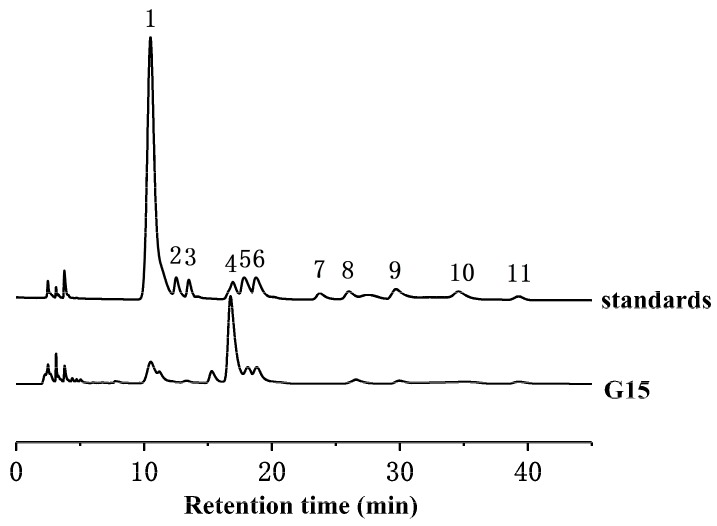
Monosaccharide composition of clam heparinoid (G15). The numbered peaks correspond to known monosaccharide standards: 1, PMP; 2, mannose (Man); 3, rhamnose (Rha); 4, glucosamine (GlcN); 5, glucuronic acid (GlcA); 6, iduronic acid (IdoA); 7, N-acetylglucosamine (GlcNAc); 8, glucose (Glc); 9, galactose (Gal); 10, arabinose (Ara), and 11, fucose (Fuc).

The monosaccharide composition of G15 was determined by the high-performance liquid chromatography (HPLC) method (Figure 3). Glucosamine was the main component of G15, accounting for 58.2%. Glucuronic acid and iduronic acid in G15 accounted for 6.25% and 12.79%, respectively. In addition, G15 contained trace amounts of glucose (3.29%), galactose (3.05%), and fucose (1.88%).

### 2.3. NMR Spectroscopy Analysis

**Figure 4:**
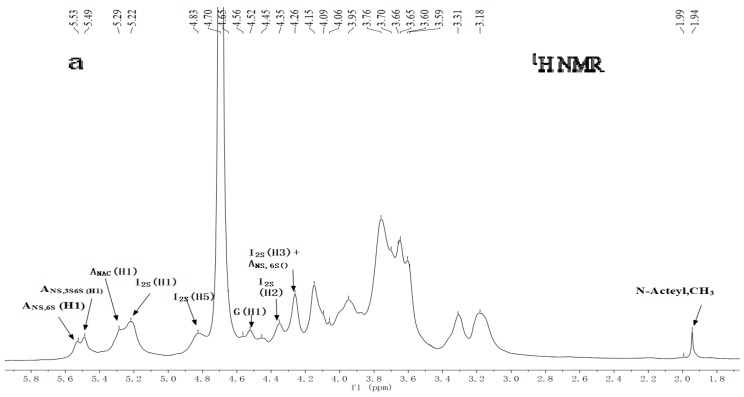

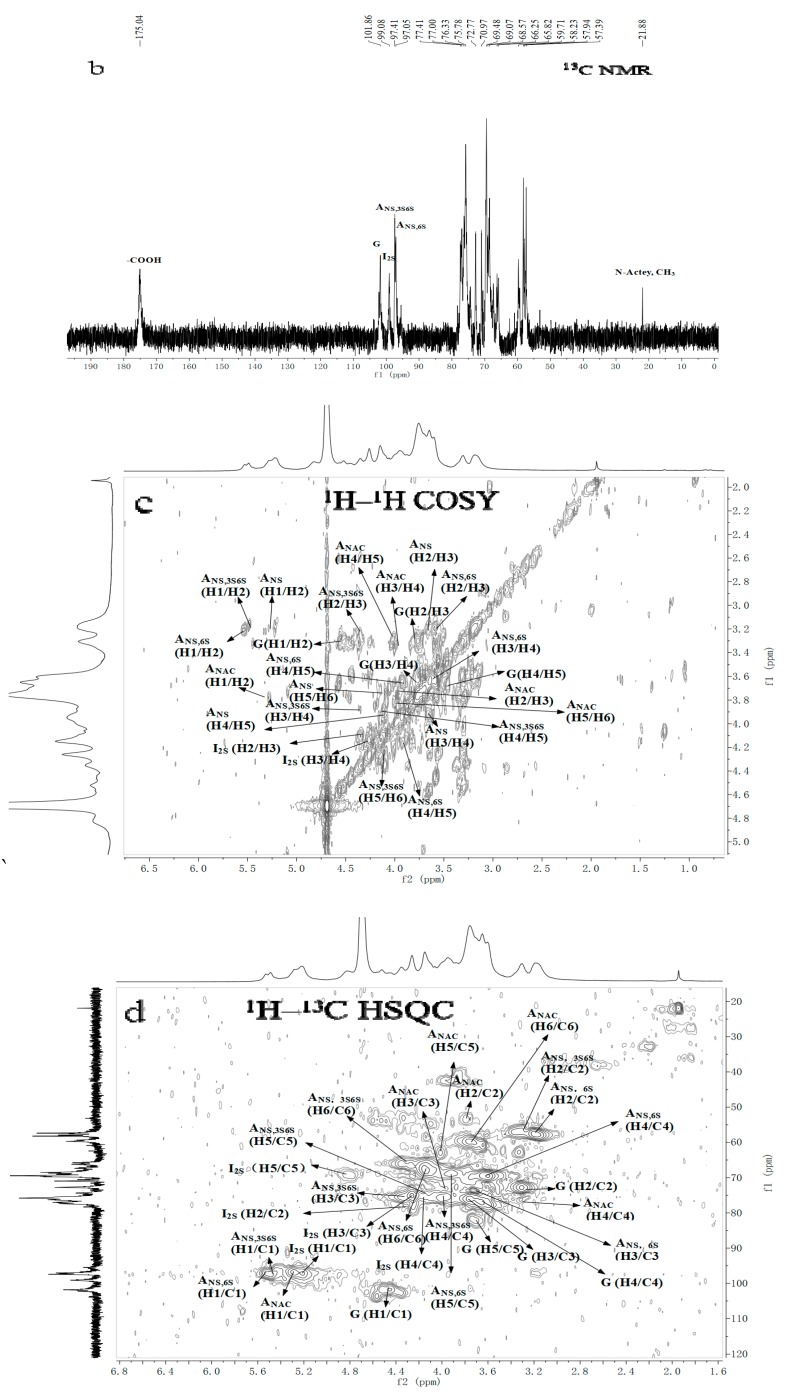

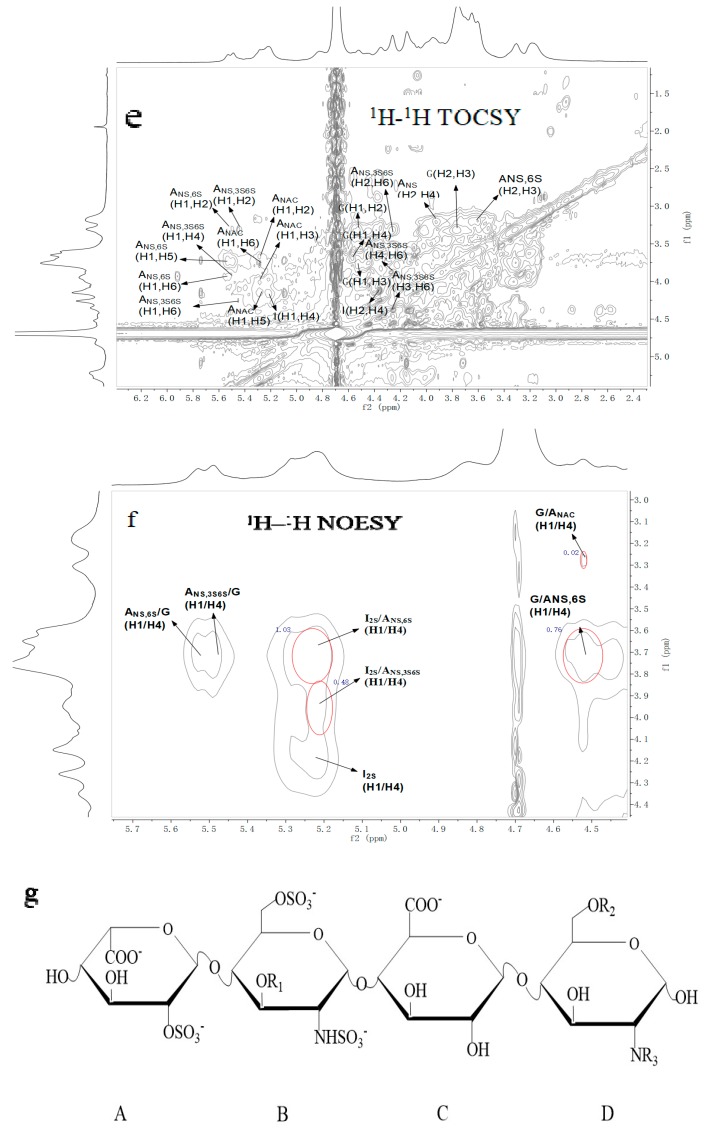
Nuclear magnetic spectra and repeat disaccharide unit of clam heparinoid (G15). (**a**) 1H NMR spectra of clam heparinoid. (**b**) 13C NMR spectra of clam heparinoid. (**c**) COSY spectra of clam heparinoid. (**d**) HSQC spectra of clam heparinoid. (**e**) TOCSY spectra of clam heparinoid. (**f**) NOESY spectra of clam heparinoid. (A_NS6__S_: N,6-disulfated glucosamine, A_NS3__S6__S_: N,3,6-trisulfated glucosamine, A_NAC_: N-acetyl glucosamine, I_2S_: 2-sulfated IdoA, and G: glucuronic acid). (**g**) Tetrasaccharide repeat unit of clam heparinoid. A = IdoA2S; B = GlcNS3S6S (R_1_ = SO_3_^-^) or GlcNS6S (R_1_ = H); C = GlcA; D = GlcNS6S (R_2_ = SO_3_^-^, R_3_ = SO_3_^-^) or GlcNAC (R_2_ = H, R_3_ = HAc).

Figure 4 shows the nuclear magnetic spectrum of G15. In the ^1^H NMR spectrum, four anomeric proton signals occurred at 5.53, 5.49, 5.29, and 5.20 ppm, which were assigned to be N,6-disulfated glucosamine (GlcNS6S), N,3,6-trisulfated glucosamine (GlcNS3S6S), N-acetyl glucosamine (GlcNAc), and 2-sulfated IdoA (IdoA2S), respectively. The signal of 4.56 ppm was attributed to GlcA. The signal of 1.9-2.2 ppm was considered to be acetamidomethyl, and the signal of 2.9-4.5 was considered to be sugar ring protons [26]. From the ^13^C NMR spectrum, the signals of 101.77, 99.08, and 97–98 ppm were corresponded to C1 of glucuronic acid, iduronic acid, and glucosamine residues, respectively. In addition, the signal of 175.04 ppm was attributed to the carboxyl group of the uronic acid residue, and the signal of 21.88 ppm corresponded to acetamidomethyl. 

The structure of G15 was further analyzed by two-dimensional NMR techniques of COSY, TOCSY, NOESY, and HSQC, and its chemical shifts were assigned based on these spectrograms and previous reports (Table 1) [27,28,29,30,31]. In general, when no group substitution occurs, the signals of C2, C3, and C4 were between 70 and 77 ppm, and the signals of C6 were between 60 and 64 ppm. When a group substitution occurs, the chemical shifts of C2, C3, and C4 move to 78–85 ppm, whereas the signals of C6 moved to 64–70 ppm [32]. The chemical shift of C2 in iduronic acid was 78.13 ppm, whereas those of C3 and C6 on the glucuronic acid residues were 78.61 and 66.74/67.30 ppm, respectively. It was believed that sulfate substitution occurred on C2 of iduronic acid and C3 and C6 of glucosamine. Moreover, several repetitive units of the sugar sequence in the heparin chain were inferred by analyzing NOESY spectra. The appearance of cross signals of H1(I2S)/H4(ANS,3S6S) indicated the linkage sequence →4)-α-IdoA2S-(1→4)-α-GlcNS3S6S-(1→ existed. The cross signal of H1(I2S)/H4(ANS,6S) suggested that the structure fragment was →4)-α-IdoA2S-(1→4)-α-GlcNS6S-(1→. The cross signal of H1(G)/H4(ANS,6S) suggested that the structure fragment was →4)-β-GlcA-(1→4)-α-GlcNS6S-(1→. And the cross signal of H1(G)/H4(ANAc) indicated the structure was →4)-β-GlcA-(1→4)-α-GlcNAc(1→. Additionally, the cross signals of H1(ANS,3S6S)/H4(G) and H1(ANS,6S)/H4(G) indicated that the tetrasaccharide structure of *C. antiquata* heparinoid was →4)-α-IdoA2S-(1→4)-α-GlcNS3S6S (or GlcNS6S)-(1→4)-β-GlcA-(1→4)-α-GlcNS6S (or GlcNAC)-(1→. Chavante et al. reported an unusual trisaccharide structure in shrimp heparin IdoA2S-(1→4)-GlcNS3S6S-(1→4) GlcA [33]. But what is the connection after GlcA in this structure? Our research may provide a reference. 

Table 1 also shows the proportion of repeating units. Among them, →4)-α-IdoA2S-(1→4)-α-GlcNS6S-(1→ accounts for the highest proportion. It is also the major disaccharide structure of mammalian heparin [34]. Compared with the previous literature [26,30,35], it is clear that the same anomeric signals are present in C. *antiquata* heparin analogues and mammalian heparin. Nevertheless, they were presented in different relative proportions.

The NMR signals attributed to the N,3,6-trisulfated glucosamine residue, a typical marker of the pentasaccharide sequence of the active site of heparin and heparan sulfates for antithrombin binding, were detected in the spectra of the clam heparin-like compound, indicating that the anticoagulant activity of such compounds may be comparable to that of heparin.

### 2.4. Anticoagulant Activity

**Figure 5:**
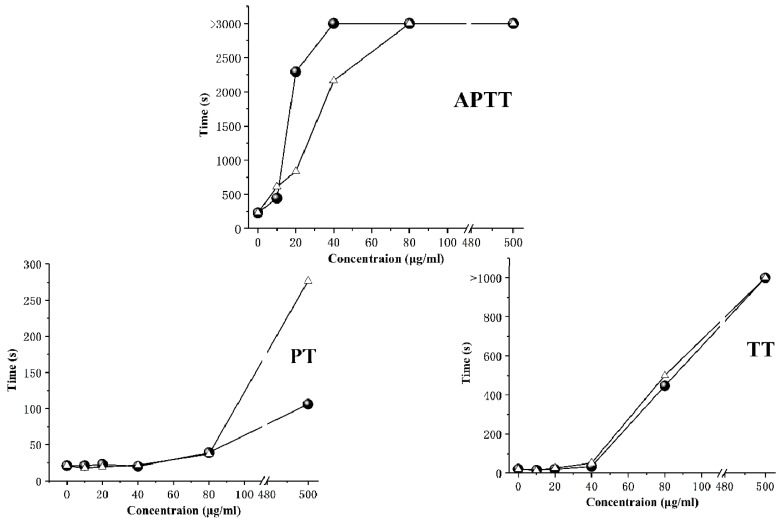
Anticoagulant activity of clam (G15) and mammalian heparins (HP) at different concentrations estimated by measuring three Indicators of Coagulation (APTT, PT, TT). ●, HP; △, G15.

The activated partial thromboplastin time (APTT), prothrombin time (PT), and thrombin time (TT) were commonly used to evaluate the anticoagulant activity of samples in medicine. APTT represents the endogenous coagulation pathway while PT corresponds to the exogenous coagulation pathway. TT represents the conversion of plasma fibrinogen to fibrin [16]. As shown in Figure 5, G15 effectively extended APTT in a concentration-dependent manner. The prolongation effect of G15 was slightly lower than that of mammalian heparins (HP). For PT, the prolongation effect of G15 beyond 500 μg/mL was about 3 times higher than that of mammalian heparins (HP). For TT, the prolongation effect of G15 was increased obviously at a concentration of more than 40 μg/mL. As a result, G15 exerted anticoagulant effects mainly through the endogenous coagulation pathway, exogenous coagulation pathway, and the inhibition of fibrinogen conversion to fibrin. The anticoagulant titer of G15 was 149.63 IU/mg, as measured by the Chinese Pharmacopoeia assay. The anticoagulant titers of crab and shrimp heparins were 33 IU/mg and 95 IU/mg, respectively, and they exert anticoagulant effects mainly through the endogenous coagulation pathway and the inhibition of plasma fibrinogen conversion to fibrin [14,36]. Compared with shrimp heparin and crab heparin, the clam heparinoid has higher anticoagulant activity and more action pathways, which may be related to its high sulfate content (21.44%) and the presence of N,3,6-trisulfated glucosamine [37].

### 2.5. Fibrinolytic Activity

#### 2.5.1. In Vitro Fibrinolytic Activity

**Figure 6:**
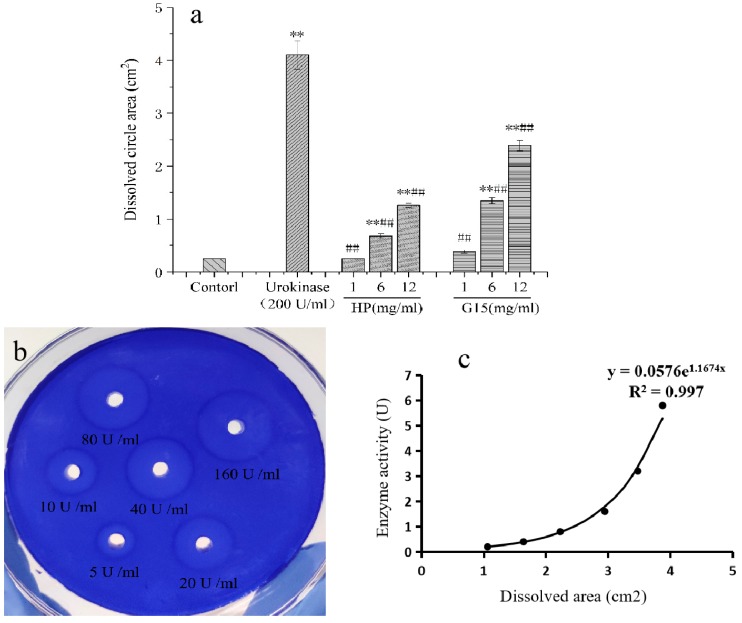
In vitro fibrinolytic activity of G15. (**a**) Dissolution ring area produced by different concentrations of G15. * *p* < 0.05, ** *p* < 0.01, as compared with the control group; # *p* < 0.05, ## *p* < 0.01, as compared with the Urokinase group. (**b**) Dissolution ring area produced by urokinase at different concentrations. (**c**) Standard curve prepared based on the urokinase concentration and dissolution ring area.

Figure 6 shows the in vitro fibrinolytic activity of G15, which is exerted in a concentration-dependent manner. The dissolved circle areas produced by G15 and HP at 6 and 12 mg/mL were significantly higher than that of the control group. Compared with the urokinase group, the dissolution rings of G15 and HP were much smaller. Besides, the dissolved circle area produced by G15 was approximately two times bigger than that of HP at 12 mg/mL (Figure 6a). Figure 6b shows the dissolved ring produced by different concentrations of urokinase to prepare the standard curve. Then, G15 and HP fibrinolytic activity was calculated to be 1.96 ± 0.11 U/mg and 0.51 ± 0.02 U/mg, respectively, based on the dissolved circle area of sample and the standard curve of urokinase (Figure 6c). Hence, G15 showed potent fibrinolytic activity in the agarose fibrin plate experiment, indicating that it may dissolve fibrin in the thrombus.

#### 2.5.2. In Vivo Fibrinolytic Activity

The effects of G15 on the fibrinolytic system in rats are shown in Table 2. Tissue-type plasminogen activator (t-PA) and urokinase-type plasminogen activator (u-PA) were the main activators in the fibrinolytic system, which could transform plasminogen into plasmin. Plasminogen activator inhibitor (PAI-1), a major regulator of the fibrinolytic system, specifically bonded with t-PA in a ratio of 1:1, resulting in the inactivation of t-PA. The ratios of t-PA and PAI-1 might reflect the total change in the fibrinolytic system [38,39]. For t-PA and u-PA, G15 effectively increased the t-PA and u-PA contents in rat serum in a concentration-dependent manner, which was similar to that with HP. As for PAI-1, G15 effectively inhibited PAI-1 release at 6 and 12 mg/kg. According to t-PA/PAI-1, G15 showed potent in vivo fibrinolytic activity in a concentration-dependent manner over the experimental dose range, whereas HP caused weak fibrinolytic activity only at 6 and 12 mg/kg. So, the fibrinolytic activity of clam heparinoid was mainly exerted by increasing t-PA and inhibiting PAI-1.

In summary, marine organism produce a large number of sulfated polysaccharides with various biological activities. In view of the increasing incidence of thrombotic diseases, effective functional foods or drugs are urgently desired. Until now, the research on anticoagulant and fibrinolytic activity of marine heparin/heparinoid is still less comprehensive than those of mammalian heparin. In the present study, anticoagulant experiments and in vivo and in vitro fibrinolysis experiments showed that the clam heparinoid exposed strong anticoagulant and fibrinolytic effects. It played an anticoagulant role in the early stage of coagulation cascade through intrinsic and extrinsic pathways, and in the later stage inhibited the formation of fibrin complex (red thrombus) through common pathways. In the body of the organism, it appeared to depress the activity of PAI-1, release t-PA and u-PA who activated proenzyme plasminogen to biologically active plasmin, as well as promote thrombus degradation. 

Although marine heparin/heparin analogues rarely enter clinical trials [40], *C. antiquata* heparinoid does show great potential in treating thrombotic patients. It may prevent thrombosis in patients through anticoagulant effects or dissolve a thrombus by fibrinolysis. An in-depth work is required to investigate the thrombolytic activity of *C. antiquata* heparinoid. We will establish a rat model of venous and arterial thrombosis to further evaluate its antithrombotic effect. Additionally, the bleeding effect will be evaluated by a mouse-tail tailing experiment.

## 3. Materials and Methods 

### 3.1. Materials

*C. antiquata* was purchased from ZhanJiang, China. Mammalian heparin (HP), chondroitin sulfate (CS), dermatin sulfate (DS), and plasminogen were obtained from the National Institutes for Food and Drug Control (China). Alkaline protease 2709 (1.2 × 105 U/g), papain (800 U/mg), rabbit plasma, thrombin (300 U/mg), urokinase (50 U/mg), and bovine plasma fibrinogen were obtained from Yuanye Biotechnology Co. Ltd. (Shanghai, China). Activated partial thromboplastin time (APTT), prothrombin time (PT), and thrombin time (TT) kits were purchased from Sun Biotech Co. Ltd. (Shanghai, China). AMBERLITE FPA98 CI macroporous anion exchange resin was purchased from Rohm and Haas (Philadelphia, PA). Standard monosaccharide references (mannose, rhamnose, glucosamine, glucuronic acid, iduronic acid, N-acetylglucosamine, glucose, galactose, arabinose, and fucose) were obtained from Sigma-Aldrich (Darmstadt, Germany). Different molecular weights of heparin standard were purchased from Adhoc International Technology Co., Ltd. (Beijing, China). All other chemicals and solvents used were of analytical grade or HPLC-grade, unless otherwise specified.

### 3.2. Animals

Female Sprague–Dawley (SD) rats (220–250 g) were housed at 23 ± 2 °C under a 12 h light/dark cycle with free access to food and water. All animal care and handling procedures were conducted in compliance with the National Institutes of Health Guide for Care and Use of the laboratory animals and approved by the Local Bioethics Committee (Guangdong Ocean University, Zhanjiang, China; document number: SYXK2014-0053).

### 3.3. Isolation and Purification of C. antiquata Heparinoid

The bivalve mollusk *C. antiquata* was acquired from the local market and immediately killed; then, soft tissue was obtained. Dried tissue was prepared by freeze-drying after degreasing with acetone. One hundred grams of this material was mixed with two volumes of 0.1 M NaCl in a blender and pH adjusted to 8.0 with NaOH, after which 5.4 mg/mL alkaline protease 2709 and 5.4 mg/mL papain were added. After incubation at 50 °C for 20 h and boiling for 10 min, the mixture was centrifuged at 8000 rpm for 15 min, after which the supernatant was applied to a 2.6 cm × 40 cm column, packed with AMBERLITE FPA98 CI and eluted with a step-wise gradient of 1.0, 1.5, and 3.5M NaCl (Figure 2A). Fraction glycosaminoglycan contents were determined by the Alcian blue assay [41]. The fraction eluted with 1.5M NaCl was collected, concentrated, and precipitated overnight at 4 °C by adding 0.4 volumes of ethanol. The recovered precipitate was solubilized in distilled water, dialyzed overnight at 4 °C, and freeze-dried. Approximately 117 mg of purified heparin was obtained from 100 g of dry shellfish flesh and designated as G15.

### 3.4. Cellulose Acetate Electrophoresis

A cellulose acetate membrane (8 cm × 12 cm) was placed in 50% methanol overnight and immersed in the electrophoretic buffer (0.1 M pyridine-0.47 M formic acid buffer, pH 3.0) for 30 min. Excess water was then removed with absorbent paper. Next, 3 mg/L G15 and reference substances (HP, CS, DS) were dropped onto the acetate fiber membrane, and electrophoresed at a constant 100 V for 35 min, stained with 0.5% (w/v) Alcian blue-acetic acid, and bleached with 2% (v/v) acetic acid [42,43].

### 3.5. Molecular Weight Analysis

G15 molecular weight was determined by high-performance gel permeation chromatography (HPGPC) on a Waters Ultrahydrogel column 500 (7.8 mm × 300 mm, Milford, MA, USA) with an Agilent 1200 refractive index detector. The sample was prepared into 5mg/mL and submitted to an isocratic elution (0.2 M Na2SO4 mobile phase) at a flow rate of 0.5 mL/min. The injection volume was 10 μL, and detector and column temperatures were set at 30 °C. The column was calibrated with a Heparin standard with known molecular weights (Mw: 3500, 5000, 8000, 15000, and 30000 Da) [44].

### 3.6. Infrared Spectroscopy Analysis

The samples were dried under an infrared lamp for 2 h, mixed with KBr, ground, and pressed into 1 mm pellets for Fourier-transform infrared (FTIR) spectral analysis in the frequency range of 4000–500 cm^−1^. The FTIR spectrum was measured on a Bruker Tensor 27 FTIR spectrometer using the OPUS 7.5 software.

### 3.7. Monosaccharide Composition Analysis

The G15 monosaccharide composition was assessed by high-performance liquid chromatography (HPLC) [45]. For this, 5 mg of sample was hydrolyzed with 2 M trifluoroacetic acid (TFA, 2 mL) at 110 °C for 6 h. After removal of TFA by co-evaporating with 2 mL methanol, the remaining material was dissolved in 1 mL distilled water. Derivatization was implemented with 100 μL hydrolysate, which was mixed with 0.3 M NaOH (200 μL) and 240 μL of 0.5 M PMP (dissolved in methanol) at 70 °C for 100 min. After cooling, the reaction was terminated with 0.3 M HCl (200 μL), the excess PMP was extracted with 2 mL chloroform, and the supernatant was filtered with a 0.2 μm-pore size syringe filter.

The sample was subjected to HPLC on an Agilent ZORBAX Eclipse XDB-C18 column (4.6 mm × 250 mm, 5 μm) (Agilent Technologies Inc., Santa Clara, CA) with a UV detection at a wavelength of 245 nm. The injection volume was 10 μL, and the detector and column temperatures were set at 30 °C. A mixture of 0.02 M phosphate (pH 6.0) and acetonitrile (83:17, v/v) was used as the mobile phase at a flow rate of 1 mL/min, and the injection volume was 10 μL. The following mono sugars were used as references: mannose, rhamnose, glucosamine, glucuronic acid, iduronic acid, glucose, galactose, and fucose.

### 3.8. NMR Spectroscopy Analysis

G15 (50 mg) was dissolved in 1 mL D2O (99.9%, Sigma-Aldrich) before analysis, and exchangeable protons were substituted by deuterium after lyophilizing three times. ^1^H, ^13^C, ^1^H-^1^H COSY, ^1^H-^13^C HSQC, ^1^H-^1^H TOCSY and ^1^H-^1^H NOESY were analyzed by an Ascend 700M spectrometer (Bruker BioSpin, Germany) at 25 °C.

### 3.9. Anticoagulant Activity

Different concentrations (10, 20, 40, 80, and 500 μg/mL) of G15 were prepared with normal saline, and HP was used as the control. The activated partial thromboplastin time (APTT), prothrombin time (PT), and thrombin time (TT) were determined following the supplier´s instructions [46,47,48].

### 3.10. Fibrinolytic Activity

#### 3.10.1. In Vitro Fibrinolytic Activity

The in vitro fibrinolytic activity of G15 and HP was measured by the agarose–fibrin plate method [49,50]. For this, 0.3 g agarose and 0.01 M phosphate buffer solution (20 mL, PBS) were mixed in a conical bottle and dissolved by microwave heating. After cooling to 55–60 °C, 10 mL fibrinogen solution (0.15%) and 1 mL thrombin solution (10 U/mL) were added to the conical flask and mixed; the mixture was then transferred to a 9 cm Petri dish and placed at room temperature for one hour. Next, 3 mm diameter holes were punched and 20 μL normal saline (control group), urokinase solution (200 U/mL), and different concentrations (1 mg/mL, 6 mg/mL, and 12 mg/mL) of G15 and HP were added to the holes. The plate was then placed in a constant temperature incubator for 18 h at 37 °C, stained with a dye solution (0.25% Coomassie brilliant blue R-250, 5% acetic acid, and 4.5% methanol), and bleached with a decolorizing solution (45% methanol, 45 % distilled water, and 10 % acetic acid). The diameter of the dissolving ring was measured with a Vernier caliper. A standard curve was prepared by the dissolved area of different active urokinases (5, 10, 20, 40, 80, and 160 U/mL), and the fibrinolytic activity of the sample was quantitatively calculated based on the dissolved area of the solution.

#### 3.10.2. In Vivo Fibrinolytic Activity 

Sprague–Dawley rats were randomly divided into five groups (6 rats/group) and fasted for 24 h before the experiment. They were anesthetized by intraperitoneal injection of chloral hydrate (0.15 mL/100 g). Different doses (1 mg/kg, 6 mg/kg, and 12 mg/kg) of G15 were prepared with normal saline, and HP was the control. Then, the samples were injected into the femoral veins of rats. After 2 h, rats were fixed in a supine position and dissected. Next, blood was collected from the abdominal aorta by vacuum sampling. The contents of t-PA, u-PA, and PAI-1 in blood were determined with an enzyme-labelling instrument (Varioskan Flash 2.4; Thermo Fisher Scientific, Waltham, MA, USA), following the manufacturer’s instructions, and then t-PA/PAI-1 was calculated [25]. 

### 3.11. Statistical Analysis

Results were expressed as the means ± standard deviation. The experimental data were subjected to an analysis of variance for a completely random design, and three samples were prepared for assays of every attribute. Data processing was performed using JMP 10, ChemDraw, and Origin 8.

## 4. Conclusions

In summary, our results indicated that purified *C. antiquata* heparinoid with a molecular weight of 30.99 kDa is mainly composed of →4)-α-IdoA2S-(1→4)-α-GlcNS3S6S (or GlcNS6S)-(1→4)-β-GlcA -(1→4)-α-GlcNS6S (or GlcNAC)-(1→. *C. antiquata* heparinoid showed a potent anticoagulant effect, which prevented the thrombosis caused by blood coagulation. To the best of our knowledge, this is the first study on the fibrinolytic activity of marine heparin, and it demonstrates that the fibrinolytic activity of marine heparin is higher than that of mammalian heparin. Hence, *C. antiquata* heparinoid shows promise for use in developing an antithrombotic agent. Additionally, it could be a very interesting alternative to mammalian heparins, with high anticoagulant and fibrinolytic activity.

## Figures and Tables

**Table 1:** ^1^H, ^13^C NMR spectral data and molar ration of repetitive units for G15.

Repetitive Units	Molar Ratio	Chemical Shifts										
		Uronic Acid					Glucosamine					
		H1	H2	H3	H4	H5	H1	H2	H3	H4	H5	H6
		C1	C2	C3	C4	C5	C1	C2	C3	C4	C5	C6
→4)-α-IdoA2S-(1→4)-α-GlcNS6S-(1→	1.03	5.20	4.34	4.25	4.14	4.83	5.53	3.21	3.60	3.65	3.92	4.17
		99.08	78.13	75.46	75.63	71.15	97.61	57.53	73.00	69.61	69.05	67.30
→4)-α-IdoA2S-(1→4)-α-GlcNS3S6S-(1→	0.48	5.20	4.34	4.25	4.14	4.83	5.49	3.2	4.35	4.00	4.13	4.27
		99.08	78.13	75.46	75.63	71.15	97.38	55.05	78.61	74.90	72.59	66.74
→4)-β-GlcA-(1→4)-α-GlcNS6S-(1→	0.76	4.56	3.31	3.76	3.67	3.71	5.53	3.21	3.60	3.65	3.92	4.17
		101.77	73.15	76.36	76.53	81.12	97.61	57.53	73.00	69.61	69.05	67.30
→4)-β-GlcA-(1→4)-α-GlcNAc(1→	0.02	4.56	3.31	3.76	3.67	3.71	5.29	3.81	3.96	3.34	4.03	3.84
		101.77	73.15	76.36	76.53	81.12	97.44	53.31	73.5	75.85	63.93	20.22

**Table 2:** Effects of G15 on the fibrinolytic system in rats.

Index	Injection	^a^ Concentration (mg/kg)			
		0	1	6	12
t-PA (ng/mL)	HP	23.86 ± 3.80	46.97 ± 5.07	65.24 ± 10.75	65.72 ± 4.23
	G15	23.86 ± 3.80	35.64 ± 4.66	41.22 ± 8.16	62.73 ± 2.66
u-PA (ng/mL)	HP	1.12 ± 0.06	1.01 ± 0.07	1.23 ± 0.09	1.41 ± 0.14
	G15	1.12 ± 0.64	1.22 ± 0.11	1.39 ± 0.24	1.58 ± 0.17
PAI-1 (ng/mL)	HP	12.80 ± 1.57	37.50 ± 17.56	33.21 ± 11.95	24.05 ± 5.93
	G15	12.80 ± 1.57	25.63 ± 6.46	9.46 ± 0.52	6.03 ± 1.74
t-PA/PAI-1	HP	1.86 ± 0.07	1.25 ± 0.68	1.96 ± 0.39	2.73 ± 0.31
	G15	1.86 ± 0.07	1.39 ± 0.44	4.35 ± 0.62	10.40 ± 1.26

^a^ Different concentrations of G15 were formulated in normal saline.
